# Exosomes in tumour immunity

**DOI:** 10.3747/co.v16i3.367

**Published:** 2009-05

**Authors:** A. Clayton, M.D. Mason

Exosomes are nanometre-size vesicles manufactured within late multi-vesicular endosomes and actively secreted into the extracellular environment^1^. The vesicles are bounded by a cholesterol-rich phospholipid membrane^2^ bearing a host of transmembrane and glycosylphosphatidylinisotol-anchored molecules^3^. Their lumen houses a cytosol-like protein repertoire, together with unique messenger rna and microrna species^4^.

In 1996, seminal work by Raposo *et al*.^1^, and soon after by Zitvogel *et al*.^5^, demonstrated that exosomes isolated from antigen-presenting cells (apcs) can act essentially as miniature antigen-presenting cell surrogates, capable of activating T cells *in vitro*—and importantly, also *in vivo*^5^. Exosomes within the extracellular milieu can therefore disseminate at least some of the parent cell functions.

## EXOSOME ACTIVATION OF T CELLS

The direct interaction of apc-derived exosomes with CD4^+^ or CD8^+^ T cells can lead to cell activation in a major histocompatibility complex (mhc)–peptide–restricted manner. Exosomes that are apc-derived must, therefore, express conformationally correct mhc–peptide complexes at the exosome surface. However, this signal delivery is also supported by key accessory factors such as exosomally expressed intracellular adhesion molecule 1 and CD80/CD86^6^.

However, more recent studies allude to the relative inefficiency of direct exosomal T-cell activation, in which the presence of dendritic cells (dcs) as a recipient surface for exosomal mhc molecules is important for enhancing the magnitude of T-cell activation^7^. *In vivo*, dc-exosomes may be further potentiated by also adding exogenous adjuvants^8^. In preclinical studies, dc-derived exosomes administered as prophylactic^9^ or therapeutic^5^ cancer vaccines demonstrated their value as potent immune-activating agents, often more so than did live dcs. Based on this premise, some phase i trials of dc-exosomes have been conducted, showing promising results in very testing clinical scenarios, such as advanced non-small-cell lung cancer^10^. Why dc-exosomes are more effective than live dcs in such therapeutic settings remains unclear. It may be that dc-exosomes can spontaneously activate host dcs or other immune cells to boost the antitumour response. Alternatively, these effects may simply be a result of the acellular nature of exosomes, capable of performing their activator functions while resisting the complex immune suppressive factors present in tumour-bearing hosts.

## CANCER EXOSOMES AND IMMUNE ACTIVATION

Cancer cells also produce exosomes, evident in culture and surprisingly abundant in malignant effusions^11^ such as peritoneal ascites of ovarian cancer^12^ and pleural fluid of mesothelioma^13^. In fact, aberrant signalling pathways, particularly those related to p53 response elements such as Steap3, may positively regulate exosome secretion, suggesting elevated exosome secretion as a property of malignancy and genotoxic stress^14^.

In many respects, cancer-derived exosomes resemble those of apc origin in their biophysical and biochemical properties. As would be expected, cell type–specific differences are also present, the most significant of which are the expression of tumour-associated antigens, particularly those found in association with the cell membrane. Comparisons of whole-tumour-cell lysates with tumour exosomes reveal often striking enrichment within the exosomes of tumour antigens such as *HER2/neu*, melan-A^11^, Silv^15^, carcinoembryonic antigen^16^, mesothelin^17^, and others. Immunization of mice with dcs pulsed with cancer cell–derived exosomes demonstrate that it is possible to induce protective antitumour immune responses using cancer-derived exosomes as a source of an antigen or antigens^15^. Similarly, in an *ex vivo* human model system, exosomes taken from malignant effusions proved an effective source of tumour antigens for cross-presentation to CD8^+^ s cytotoxic T cells by dcs^11^. This aspect has since been explored in the context of phase i studies^12^,^18^, albeit with one or more added factors for enhancing or recruiting dc functions. To date, direct activation of T cells by cancer exosomes has not been shown; rather the T-cell stimulatory function of cancer exosomes requires uptake and processing by professional apcs, which subsequently elicit T-cell activation.

Of notable interest, however, is the suggestion that cancer exosomes do not act as a passive form of antigen; on the contrary, such exosomes may be superior to other forms of antigen such as whole-cell lysates^15^ or soluble antigen^19^. This may be the result of an advantageous delivery of antigen in the form of exosomes, which may bind and be taken up efficiently by dcs. The molecule Mfg-E8 (lactadherin) expressed by dc-exosomes^3^,^20^ has been implicated in the interaction between dc-exosomes and dcs^21^. This molecule is not necessarily involved in the binding and uptake of cancer exosomes. Molecules such as integrins^22^, tetraspanins, and others^21^ have been implicated in exosome–adhesion interactions, but the key to this apparent advantageous targeting of cancer exosomes to dcs remains elusive. Expression of heat shock proteins (such as Hsp70) at the exosome surface may be an interesting candidate, not only as a cofactor for efficient receptor-mediated uptake, but also for imparting “danger” signals that trigger dc maturation and that subsequently enhance immunologic activation. Thus, exposing cancer cells to stress may render their exosomes significantly more immunogenic^16^,^23^. These activities require the active participation of dcs in processing and in cross-presenting exosomally delivered antigens, but it is important to emphasize that the cancer exosome phenotype, which is under the influence of micro-environmental factors, is important for these immune functions.

Stress proteins expressed on the surface of cancer cell–derived exosomes may also have influence over other cell types, and are therefore not dc-selective. Gastpar *et al*. nicely showed that Hsp70 present at the exosome surface (from colorectal cancer cell lines) could directly activate natural killer (nk) cells, supporting migration and cytotoxic functions. In contrast, sub-lines that produced exosomes lacking surface Hsp70 were poorly activating^24^. Exosomal Hsp expression is a complex issue; and even when elevated exosomal Hsp expression is apparent after stress, the elevation may not always correlate with enhanced immune function—a difference attributable to luminal as compared with surface expression of Hsp^25^.

## CANCER EXOSOMES AND IMMUNE SUPPRESSION

We have cited several examples of cancer exosomes exerting a positive influence on the immune system, but these scenarios do not seem to be well reflected in the clinical setting. We know that patients with gross malignant ascites produce copious quantities of exosomes *in vivo*^11^,^12^. Yet, regardless of the exosome content of such fluids, the disease more often than not pursues a progressive course. Anecdotally, therefore, the concept of natural immune-activating cancer exosomes may be misleading, at least in an advanced disease setting. An alternative view suggests that the secretion of vesicles that would encourage immune-mediated destruction of the tumour is not in a cancer cell’s interest. It is more likely, in our view, that cancer exosomes reflect the aims and functions of the parent cancer cell: that is, to survive, grow, and metastasize—and some available evidence supports this view^26^,^27^. Is it possible that cancer exosomes also act to assist cancers in immune evasion?

Mounting evidence is indeed pointing to exosomes as major participants in immune evasion. Although the concept of tolerance induced by exosomes was well described in the context of acquired dietary antigens^28^–^30^ and, more recently, in reproductive biology^31^, transplantation^32^, and respiratory allergens^33^, several novel mechanisms (both direct and indirect) have recently been described in the context of cancer exosomes.

Among the earliest such reports is a description of melanoma-derived exosomes that were lethal to T cells^34^. These cancer cells naturally express Fas ligand, and expel by the multivesicular endosomal route at least a proportion of this molecule in the form of exosomes. FasL–bearing exosomes, upon encountering activated (Fas-positive) T cells, can essentially crosslink T cell Fas and trigger apoptotic death^34^. Other influences of exosomally expressed members of the tumour necrosis factor superfamily may include downmodulation by ovarian cancer exosomes of the CD3-ζ chain. This molecule is an integral component of the T-cell receptor (tcr) complex, which is essential for competent signalling after tcr–mhc–peptide interactions^35^. Melanoma exosomes expressing tumour necrosis factor α may also affect the CD3–tcr complex in a reactive oxygen species–mediated manner^36^. Thus, cancer exosomes can exert drastic effects to oppose one or more T-cell functions and, in some situations, may constitute an important mechanism by which tumours eliminate activated T cells that may recognize and kill them^34^.

However, apoptotic death of T cells is not a universal consequence of interactions with exosomes. The outcome depends both on T-cell status and on the molecular phenotype of the exosome. In chronic inflammatory disease, for example, exosomes may in fact attenuate T-cell apoptosis, prolonging their survival inappropriately and adding to persistent inflammatory injury^37^. Other death-independent effects of cancer exosomes on the immune system have been reported. Liu *et al*., for example, pre-treated mice with breast cancer exosomes before implanting tumours and documented accelerated tumour growth^38^. This accelerated growth was result of the negative influence of cancer exosomes on nk cell functions, inhibiting nk cell proliferation (in response to interleukin-2) and impairing subsequent cytotoxic functions. Similarly, studies by other researchers showed that human nk cells also become significantly functionally impaired following treatment with several cancer exosome types, manifested 2 by downmodulation of nkg2d^39^, which is among the most important tumour-recognition molecules for nk cells. This molecule is also of importance for other lymphocyte subsets, such as CD8^+^ T cells, γδ–T cells, nk–T cells, and others. Cancer exosomes may therefore negatively modulate the functions of multiple branches of the immune system, with effects seemingly particularly focussed toward suppressing cytotoxic function.

Many of the cellular responses described above may well occur through direct interactions between immune effector cells and cancer exosomes, although the molecular participants are not entirely understood in each case. However, evidence is also available to suggest that cancer exosomes may be exploiting the regulatory arms of immunity and thus exerting their effects indirectly. One example describes the induction of human regulatory T cells (CD4^+^CD25^+^Foxp3^+^) by mesothelioma-derived exosomes, which thereafter exert dominant antiproliferative effects on lymphocyte responses to interleukin-2^17^. The mechanism for this effect was MHC class II–independent, relying instead on exosomally expressed transforming growth factor β1^17^.

A robust antitumour response relies not only on competent effector cells, it also heavily depends on functional apcs. Here again, there are examples in which cancer exosomes mediate negative effects. The differentiation of dcs from bone marrow precursors (murine system) or from monocytes (humans) becomes severely impaired in the presence of tumour exosomes^40^, largely because of exosomal induction of interleukin-6 expression by precursor cells. Similar examples suggest that tumour exosomes not only interfere with dc differentiation, but actively skew precursors toward acquisition of a myeloid suppressor cell phenotype^41^. In turn, such cells mediate negative regulation of effector cells through the secretion of soluble transforming growth factor β.

Cancer-derived exosomes can therefore target both the effector and the antigen-presentation arms of the immune system. Whether exosomes from a given tumour harbour sufficient complexity to be capable of a multitude of suppressive mechanisms is not yet known. Nevertheless, the many mechanisms described to date for several cancer exosome types highlight exosomes as a major tool for immune evasion.

## SUMMARY

Exosomes secreted by cancer cells are dynamic and highly complex, and the field as it stands remains somewhat controversial. It may be that, in early neoplastic lesions, cancer cells and the exosomes they produce have not yet acquired the potent suppressive molecules and mechanisms described here. Under such conditions, exosomes may play an important role in disseminating relevant tumour rejection antigens to the immune system, assisting the immune response, through the activities of dcs. However, by its very existence, progressive disease has overcome or overwhelmed the immune response, and exosomes in these scenarios harbour multiple mechanisms for attenuating several branches of immunity.

Identifying the factor or factors responsible for this possible switch from immunogenic to immune-suppressive exosomes will be a major challenge, but will in turn offer exciting novel therapeutic opportunities for blocking tumour immune escape while retaining efficient tumour-antigen handling by the immune system.

## References

[1] Raposo G, Nijman HW, Stoorvogel W (1996). B Lymphocytes secrete antigen-presenting vesicles. J Exp Med.

[2] Wubbolts R, Leckie RS, Veenhuizen PT (2003). Proteomic and biochemical analyses of human B cell–derived exosomes. Potential implications for their function and multivesicular body formation. J Biol Chem.

[3] Théry C, Regnault A, Garin J (1999). Molecular characterization of dendritic cell–derived exosomes. Selective accumulation of the heat shock protein Hsc73. J Cell Biol.

[4] Valadi H, Ekström K, Bossios A, Sjöstrand M, Lee JJ, Lötvall JO (2007). Exosome-mediated transfer of mrnas and micrornas is a novel mechanism of genetic exchange between cells. Nat Cell Biol.

[5] Zitvogel L, Regnault A, Lozier A (1998). Eradication of established murine tumors using a novel cell-free vaccine: dendritic cell–derived exosomes. Nat Med.

[6] Hwang I, Shen X, Sprent J (2003). Direct stimulation of naive T cells by membrane vesicles from antigen-presenting cells: distinct roles for CD54 and B7 molecules. Proc Natl Acad Sci U S A.

[7] André F, Chaput N, Schartz NE (2004). Exosomes as potent cell-free peptide-based vaccine. I. Dendritic cell–derived exosomes transfer functional mhc class I/peptide complexes to dendritic cells. J Immunol.

[8] Chaput N, Schartz NE, André F (2004). Exosomes as potent cell-free peptide-based vaccine. II. Exosomes in CpG adjuvants efficiently prime naive Tc1 lymphocytes leading to tumor rejection. J Immunol.

[9] Altieri SL, Khan AN, Tomasi TB (2004). Exosomes from plasmacytoma cells as a tumor vaccine. J Immunother.

[10] Morse MA, Garst J, Osada T (2005). A phase i study of dexosome immunotherapy in patients with advanced non-small cell lung cancer. J Transl Med.

[11] Andre F, Schartz NE, Movassagh M (2002). Malignant effusions and immunogenic tumour-derived exosomes. Lancet.

[12] Navabi H, Croston D, Hobot J (2005). Preparation of human ovarian cancer ascites-derived exosomes for a clinical trial. Blood Cells Mol Dis.

[13] Bard MP, Hegmans JP, Hemmes A (2004). Proteomic analysis of exosomes isolated from human malignant pleural effusions. Am J Respir Cell Mol Biol.

[14] Yu X, Harris SL, Levine AJ (2006). The regulation of exosome secretion: a novel function of the p53 protein. Cancer Res.

[15] Wolfers J, Lozier A, Raposo G (2001). Tumor-derived exosomes are a source of shared tumor rejection antigens for CTL cross-priming. Nat Med.

[16] Dai S, Wan T, Wang B (2005). More efficient induction of HLA-A*0201-restricted and carcinoembryonic antigen (cea)–specific ctl response by immunization with exosomes prepared from heat-stressed cea-positive tumor cells. Clin Cancer Res.

[17] Clayton A, Mitchell JP, Court J, Mason MD, Tabi Z (2007). Human tumor-derived exosomes selectively impair lymphocyte responses to interleukin-2. Cancer Res.

[18] Dai S, Wei D, Wu Z (2008). Phase i clinical trial of autologous ascites-derived exosomes combined with gm–csf for colorectal cancer. Mol Ther.

[19] Zeelenberg IS, Ostrowski M, Krumeich S (2008). Targeting tumor antigens to secreted membrane vesicles *in vivo* induces efficient antitumor immune responses. Cancer Res.

[20] Véron P, Segura E, Sugano G, Amigorena S, Théry C (2005). Accumulation of Mfg-E8/lactadherin on exosomes from immature dendritic cells. Blood Cells Mol Dis.

[21] Morelli AE, Larregina AT, Shufesky WJ (2004). Endocytosis, intracellular sorting, and processing of exosomes by dendritic cells. Blood.

[22] Clayton A, Turkes A, Dewitt S, Steadman R, Mason MD, Hallett MB (2004). Adhesion and signaling by B cell–derived exosomes: the role of integrins. FASEB J.

[23] Chen W, Wang J, Shao C (2006). Efficient induction of antitumor T cell immunity by exosomes derived from heat-shocked lymphoma cells. Eur J Immunol.

[24] Gastpar R, Gehrmann M, Bausero MA (2005). Heat shock protein 70 surface-positive tumor exosomes stimulate migratory and cytolytic activity of natural killer cells. Cancer Res.

[25] Clayton A, Turkes A, Navabi H, Mason MD, Tabi Z (2005). Induction of heat shock proteins in B-cell exosomes. J Cell Sci.

[26] Gesierich S, Berezovskiy I, Ryschich E, Zöller M (2006). Systemic induction of the angiogenesis switch by the tetraspanin D6.1A/CO-029. Cancer Res.

[27] Hao S, Ye Z, Li F (2006). Epigenetic transfer of metastatic activity by uptake of highly metastatic B16 melanoma cell–released exosomes. Exp Oncol.

[28] Karlsson M, Lundin S, Dahlgren U, Kahu H, Pettersson I, Telemo E (2001). “Tolerosomes” are produced by intestinal epithelial cells. Eur J Immunol.

[29] van Niel G, Raposo G, Candalh C (2001). Intestinal epithelial cells secrete exosome-like vesicles. Gastroenterology.

[30] Mallegol J, van Niel G, Heyman M (2005). Phenotypic and functional characterization of intestinal epithelial exosomes. Blood Cells Mol Dis.

[31] Taylor DD, Akyol S, Gercel–Taylor C (2006). Pregnancy-associated exosomes and their modulation of T cell signaling. J Immunol.

[32] Pêche H, Renaudin K, Beriou G, Merieau E, Amigorena S, Cuturi MC (2006). Induction of tolerance by exosomes and short-term immunosuppression in a fully mhc-mismatched rat cardiac allograft model. Am J Transplant.

[33] Prado N, Marazuela EG, Segura E (2008). Exosomes from bronchoalveolar fluid of tolerized mice prevent allergic reaction. J Immunol.

[34] Andreola G, Rivoltini L, Castelli C (2002). Induction of lymphocyte apoptosis by tumor cell secretion of FasL-bearing microvesicles. J Exp Med.

[35] Taylor DD, Gerçel–Taylor C (2005). Tumour-derived exosomes and their role in cancer-associated T-cell signalling defects. Br J Cancer.

[36] Söderberg A, Barral AM, Söderström M, Sander B, Rosén A (2007). Redox-signaling transmitted in trans to neighboring cells by melanoma-derived tnf-containing exosomes. Free Radic Biol Med.

[37] Zhang HG, Liu C, Su K (2006). A membrane form of tnf-α presented by exosomes delays T cell activation–induced cell death. J Immunol.

[38] Liu C, Yu S, Zinn K (2006). Murine mammary carcinoma exosomes promote tumor growth by suppression of nk cell function. J Immunol.

[39] Clayton A, Mitchell JP, Court J, Linnane S, Mason MD, Tabi Z (2008). Human tumor-derived exosomes down-modulate *NKG2D* expression. J Immunol.

[40] Yu S, Liu C, Su K (2007). Tumor exosomes inhibit differentiation of bone marrow dendritic cells. J Immunol.

[41] Valenti R, Huber V, Filipazzi P (2006). Human tumor-released microvesicles promote the differentiation of myeloid cells with transforming growth factor-β–mediated suppressive activity on T lymphocytes. Cancer Res.

